# A novel solvent-free co-grinding preparation improves curcumin bioavailability in healthy volunteers: A single-center crossover study

**DOI:** 10.1016/j.heliyon.2023.e12829

**Published:** 2023-01-05

**Authors:** Chenjing Wang, Xin Jiang, Xiaolei Zhang, Yi Xu, Li Li, Xin Li, Shanglong Wang, Ping Shi, Xiaomeng Gao, Zimin Liu, W. Dennis Clark, Yu Cao

**Affiliations:** a Phase I Clinical Research Center, The Affiliated Hospital of Qingdao University, No.16 Jiangsu Road, Shinan District, Qingdao City, Shandong Province 266003, China; b Chenland Research Institute, 333 Songling Road, Laoshan District, Qingdao City, Shandong Province 266104, China; c Chenland Nutritionals, Inc., 3 Park Plaza, Suite 0410, Irvine, CA 92614, USA

**Keywords:** Curcumin, Oral bioavailability, Pharmacokinetics, CUMINUP60®

## Abstract

Curcumin, from the rhizome of turmeric (*Curcuma longa* L.), has a wide variety of biological activities. Unfortunately, its poor water-solubility greatly limits its bioavailability. The purpose of this study was to evaluate CUMINUP60®, a novel preparation utilizing a solvent-free, co-grinding method designed to improve curcumin’s bioavailability. We performed a single-center crossover experiment to compare the new product with standard 95% curcumin in the blood plasma of twelve healthy adults (10 males, 2 females). Total bioavailability of curcumin and its sulfate and glucuronide conjugates from the test product, measured by their areas under the curve over 12 h (AUC_0-T_), showed a combined increase of 178-fold over standard curcumin and its conjugates from the reference product. The new product represents a significant improvement for providing greater bioavailability of curcumin, as compared with several other branded preparations. It therefore has broad applications for preparing curcumin as a more effective health ingredient in functional foods, beverages, and nutraceuticals.

## 1 Introduction

Curcumin is the main bioactive component from the rhizomes of turmeric (*Curcuma longa* L.). Numerous human clinical and animal studies have shown the health benefits of curcumin. Historically they include antioxidant, anti-inflammatory, immunoprotective, antimicrobial, antineoplastic, antidepressant, and neuroprotective activities [1,2,3,4,5,6,7,8]. More recently curcumin has also been explored as a treatment for neuropsychiatric and neurodegenerative diseases [9,10,11,12].

The molecular mechanisms behind curcumin’s broad spectrum of activities reveal its influence on a wide variety of targets, including multiple transcription factors and a host of enzymes and other gene products [8,13].

Despite its potential benefits, the application of curcumin as a therapeutic agent suffers from its extremely low oral bioavailability. Several strategies have been developed for increasing absorption [14,15,16,17,18,19,20,21,22,23,24,25]. However, their commercial applications can be hampered by overly complicated processing, high cost, low ingredient-loading capacities, gastric irritation, and the necessity of using environmentally unfriendly organic solvents.

To overcome these problems, a simple process was developed using a solvent-free co-grinding method to improve the bioavailability of curcumin with high loading capacity. This preparation boosted cellular bioavailability in three human colon cancer cell lines (Caco-2, HT-29, human Burkitt’s lymphoma) and in male Sprague-Dawley rats [26].

In addition to evaluating free curcumin, plasma analyses also followed the concentrations of two extensively transformed curcumin conjugates, glucuronide and sulfate, known in both rodents and in humans [27,28,29]. Results revealed that the preparation manufactured by the new delivery system, CUMINUP60®, improved total bioavailability of curcumin and its conjugates several-fold over that of standard curcumin.

The main aim of the present study was to evaluate the bioavailability and plasma pharmacokinetics of this novel oral delivery system in human subjects in comparison with standard 95% curcumin.

## 2 Materials and methods

### 2.1 Curcumin study products

The test product used in this study, CUMINUP60®, was developed and supplied by Qingdao Chenland Pharmaceutical Technology Development Co., Ltd. (Qingdao City, Shangdong Province, China). The preparation method was first reported by Ref. [26]. For the current study it entailed combining one part of the excipient, Kolliphor® Poloxamer 407 (BASF, Shanghai, China), with two parts of a turmeric rhizome extract containing 95% curcumin. Two small steel balls were added to the mixture, which was then placed in a grinder for grinding at a frequency of 60 Hz for 2 min (Automatic Grinding Instrument (JXFSTPRP24, Shanghai Jingxin Industrial Development Co., Ltd.).

The test product, composed of 65–70% extract and 30–35% excipient, was provided for the study as 400 mg capsules (Batch No. JHS-20210102). The reference product, 95% curcumin, came from the same source (Batch No. JHS-20210103), also in 400 mg capsules.

### 2.2 Human subjects

This study was approved by the Ethics Committee of the National Drug Clinical Trial Institution of Affiliated Hospital of Qingdao University on February 24, 2021, with approval number QYFYEC 2021-014-01. The ethics review and approval process strictly abided by the ethical principles of human medical research in the Declaration of Helsinki, Good Clinical Practice issued by NMPA, ICH/GCP and corresponding requirements of domestic laws and regulations. The study was performed from April 16, 2021, to April 23, 2021, at the National Drug Clinical Trial Institution of Affiliated Hospital of Qingdao University, Shandong Province, China. All volunteers signed the written informed consent form in duplicate, one retained by the study center and the other retained by the subject.

Subjects of both genders were included in the study once they understood the content, processes, and possible adverse reactions from the test product, then signed the informed consent form. Additional inclusion criteria included being able to complete the study as required by the protocol; having taken effective contraception (male and female) within 14 days prior to screening; willing to continue taking contraception voluntarily, without planning a pregnancy within 3 months after the completion of the study; being between the ages of 18–55 years (inclusive); weighing no less than 50 kg (male) or 45 kg (female); and having a BMI between 18.0 and 28.0 (inclusive).

Subjects with the following conditions were excluded from the study: having clinically significant abnormalities as judged by the clinician, including a history of heart, liver, kidney, digestive tract, nervous system, respiratory system, mental disorders, and metabolic disorders; abnormalities based on the physical examination (ECG, clinical laboratory testing, vital signs); testing positive for hepatitis B surface antigen, hepatitis C antibody, HIV antibody, syphilis antibody, or COVID-19 antibody; having a history of specific allergies to pollen, milk or other foods, to two or more drugs, or to curcumin components or analogues; drinking more than 14 units of alcohol per week (1 unit = 285 mL of beer, 25 mL of spirits, or 100 mL of wine); having a history of dysphagia or any gastrointestinal disorder affecting drug absorption (e.g., gastric or small bowel resection, atrophic gastritis, gastrointestinal bleeding, obstruction; being pregnant or lactating; screening positive for drugs, having used drugs in within 3 months prior to the study, or having a history of drug abuse; smoking more than 5 cigarettes per day, on average, within 3 months prior to the study; donating or losing more than 400 mL of blood within 3 months prior to the study, or donating 2 therapeutic doses of platelets (1 therapeutic dose = 12 units of platelets) within 1 month prior to the study; having surgery within 3 months prior to the study; having taken curcumin within 3 months prior to the study; having taken any prescription drug within 14 days prior to the study; having taken any over-the-counter drug, herbal medicine, or other health product within 7 days prior to the study; consuming grapefruit or grapefruit juice, ginger, or curry within 48 h prior to the study; consuming chocolate or any other caffeinated or xanthine-rich drinks or foods (e.g., liver) within 48 h prior to the study undergoing strenuous exercise within 48 h prior to the study; consuming or screening positive for alcohol within 24 h prior to the study.

Twelve healthy subjects were enrolled under the protocol (Table 1). Prior to enrollment in the study, all volunteers were made fully aware of the purpose, content and process of the study, the benefits and potential risks of participating in the study, and were informed of that they should participate in the study voluntarily and could be withdrawn from the study at any time and for any reason.

**Table 1:** Demographic data of human subjects.

Subject	Height (cm)	Weight (kg)	BMI (kg/m^2^)	Age	Gender
1001	180.00	69.00	21.30	22	Male
1002	174.00	55.00	18.17	19	Male
1003	178.50	61.00	19.14	23	Male
1004	169.00	59.00	20.66	27	Male
1005	177.00	79.00	25.22	44	Male
1006	164.50	64.50	23.84	23	Female
1007	175.00	67.00	21.88	21	Male
1008	175.00	66.00	21.55	25	Male
1009	174.00	80.00	26.42	24	Male
1010	163.50	63.50	23.75	44	Female
1011	175.00	64.50	21.06	33	Male
1012	176.00	72.50	23.41	20	Male
	173.46 ± 5.17^a^	66.75 ± 7.17^a^	22.20 ± 2.31^a^	27.08 ± 8.33^a^	

a x̄ ± SD.

The safety of the test product and the reference product was assessed by the incidences of adverse events, laboratory test results (hematology, urinalysis, and blood biochemistry), vital signs evaluation, ECG results, and physical examination results.

One male subject withdrew before completing the second phase of the study.

### 2.3 Study design

Subjects were evenly divided into two groups. All subjects in both groups began on Day 1 after an overnight fast of at least 10 h.

In Period 1, those in one group each received 4 capsules (1600 mg) of the test product. Those in the other group each received 4 capsules (1600 mg) of the reference product. Subjects took their corresponding samples with 240 mL warm water.

Venous blood samples (4 mL ea.) were taken at 0 h (within 60 min before product administration) at 0.25 h, 0.5 h, 1.0 h, 1.5 h, 2.0 h, 2.5 h, 3.0 h, 3.5 h, 4.0 h, 4.5 h, 5.0 h, 6.0 h, 8.0 h, 10.0 h, 12.0 h, 24.0 h, 36. o h, 48.0 h, and 72.0 h after administration.

Vital signs (including body temperature, pulse and blood pressure) were measured and blood was collected for lab testing at 0 h of administration (within 60 min before administration), 4 h (±30 min), 12 h (±30 min), 24 h (±60 min), 48 h (±60 min) and 72 h (±60 min) after administration.

Lab testing included complete CBC analysis (white blood cell count with differential [neutrophils, lymphocytes, monocytes, eosinophils, basophils], red blood cell count, hemoglobin, hematocrit, platelet count, red cell blood count indices [mean corpuscular volume, mean corpuscular hemoglobin, mean corpuscular hemoglobin concentration, red cell distribution width, mean platelet volume]), liver function (ALT, AST, ALP, total bilirubin), kidney function (creatinine, electrolytes [Na^+^, K^+^, Cl^−^, eGFR), TSH, and glucose.

Subjects remained in the Clinical Study Center until after blood collections and vital sign examinations were completed and approved through the 72 h period after administration. The potential influence of diet was minimized during the 72-h sampling periods of the study by requiring all subjects to consume the same meals provided by the Clinical Study Center.

Prior to leaving the center, subjects were informed by staff of the washout period precautions and informed to report any adverse events occurring during the washout period.

Following a 7-day washout period after the initial administration, subjects returned to the Clinical Study Center for Period 2, beginning on Day 8 of the study. Vital signs were taken again before Period 2 began. Protocols in Period 2 were identical to those of Period 1. The group taking the test product in Period 1 switched to the reference product for Period 2, while the second group switched to the test product. Subjects were allowed to leave the study after completing safety examinations 72 h after initial administration in Period 2.

### 2.4 Plasma sample preparation

Each sample consisted of 4 mL of venous whole blood collected into an anticoagulant tube containing EDTA-K_2_. Samples were collected under a yellow fluorescent lamp throughout the study. Within 30 min of collection, each sample tube was gently inverted 5–6 times and placed in an ice bath before being transferred to a pre-cooled low-temperature centrifuge set at 4 °C. Centrifugation was at 1700×*g* for 10 min. Aliquots (800 μL) of plasma samples were then put into pre-cooled cryogenic tubes, which were placed within 1 h into a −20 °C freezer, then transferred within 24 h to a −80 °C freezer for longer term storage.

Prior to analysis, samples were thawed in wet ice and briefly vortexed before aliquots were transferred to a 96-well deep hole plate.

For free curcumin analyses, 100 μL aliquots were mixed with 25 μL of a formate/MeOH solution (10 mM NH_4_HCO_2_ adjusted to pH 3.0 with HCO_2_H, in 100% MeOH, 1:4 v/v) and 50 μL of pH 3.0 NH_4_HCO_2_ buffer, followed by vortexing with 600 μL (CH_3_)_3_COCH_3_ and centrifuged for 15 min at 1700×*g* at 4 °C.

Aliquots (300 μL) of supernatants were transferred to a 96-well deep hole plate, air-dried with nitrogen, then swirled for 5 min with 150 μL of the formate/MeOH solution and stored at 4 °C prior to analysis.

For curcumin conjugate analyses, 50 μL aliquots were combined with 25 μL of the formate/MeOH solution and eddy mixed for 3 min, followed by 5 min vortexing with 400 μL of 0.1% HCO_2_H in CH_3_CN and 15 min centrifugation at 1700×*g* at 4 °C.

Aliquots (200 μL) of supernatants were transferred to a 96-well deep hole plate, air-dried with nitrogen, then swirled for 5 min with 200 μL of the formate/MeOH solution and stored at 4 °C prior to analysis.

### 2.5 Analytical methods

Liquid chromatography was performed on a Shimadzu HPLC system (Shimadzu, Tokyo, Japan) consisting of an SCL-10A system controller, two LC-10AD pumps, and an SIL-10AD autosampler. Quantitation was carried out by a Triple Quad 6500 MS/MS system (AB Sciex Pte. Ltd., Singapore) using negative electrospray ionization in multiple-reaction-monitoring (MRM) mode.

The column used for free curcumin was an ACE C18 (2.1 × 100 mm, 5.00 μm) from Advanced Chromatography Technologies Ltd. (Hyderabad, India). Pump A was 0.02% CH_3_CO_2_H and pump B was 0.1% HCO_2_H in CH_3_CN. Initial conditions were 40% pump B, increasing linearly to 60% at 1.30 min, to 90% at 1.40 min, and returning to 40% from 2.00 to 2.10 min.

For curcumin conjugate analyses, the column was an Ultimate XB C18 (2.1 × 100 mm, 5.00 μm) by Welch Materials, Inc. (Shanghai, China).

Pump A was 0.02% CH_3_CO_2_H with 1 mM NH_4_CH_3_CO_2_ and pump B was 0.01% CH_3_CO_2_H in CH_3_CN. Initial conditions were 25% pump B, increasing linearly to 90% at 1.80 min, and returning to 25% from 2.40 to 2.50 min.

Columns for conjugate analyses were heated to 35 °C. Flow rate was 1.0 mL/min. Total cycle times between samples were about 3 min.

HPLC analyses were calibrated using curcumin-d_6_ as the internal standard.

### 2.6 Pharmacokinetic analysis

Pharmacokinetic parameters were calculated by adopting a noncompartmental model and actual blood sampling time points with SAS V9.4 software. The main parameters were evaluated as times to peak plasma concentration (T_max_), maximum plasma concentrations (C_max_), and areas under the concentration-time curve (AUC) from time zero to the last sample collection time (T) at which the concentration can be accurately determined (AUC_0-T_).

AUC was determined by the trapezoid rule [30].

### 2.7 Bioavailability analysis

Percent bioavailability (F%) of each constituent was calculated as AUC_0-T_ from the test product, divided by that of the reference product, x 100. Total bioavailability was determined by calculating F% for the sum of all constituents.

## 3 Results

### 3.1 Pharmacokinetics

**Fig. 1:**
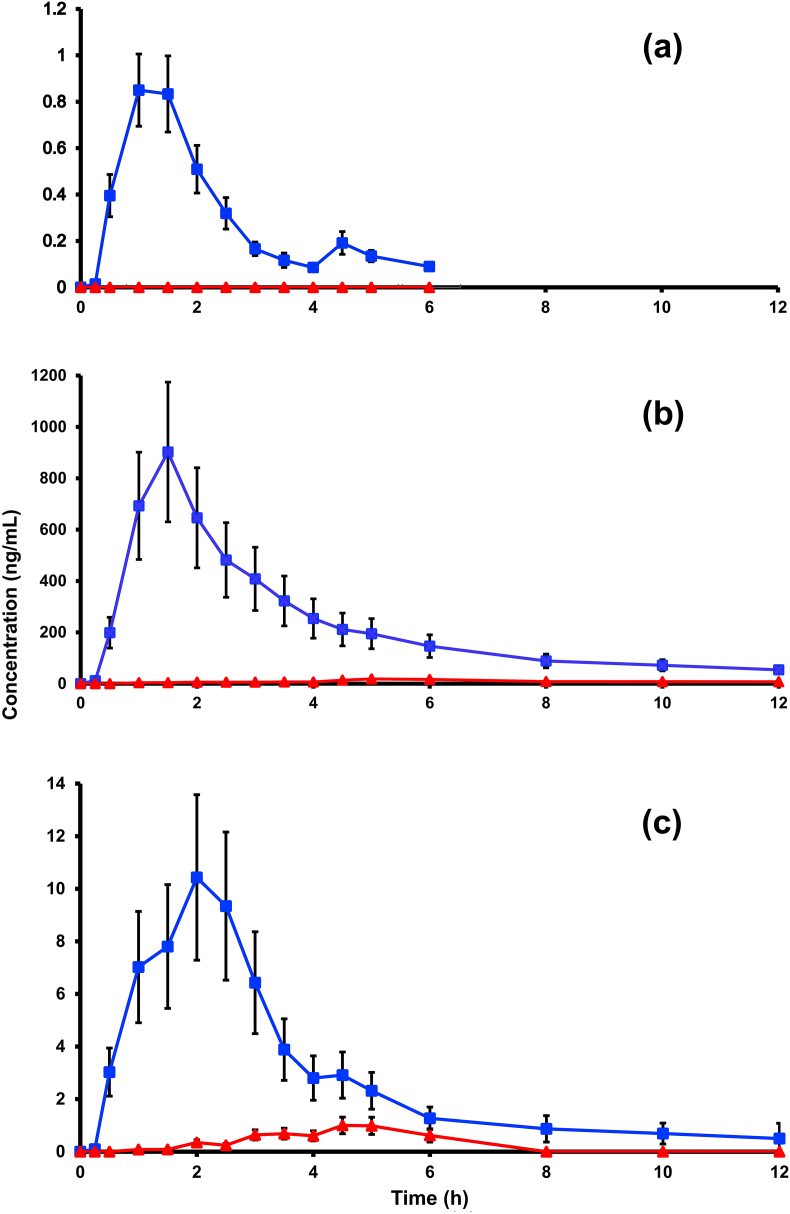
Plasma levels of free curcumin (**a**), curcumin sulfate (**b**), and curcumin glucuronide (**c**) after a single dose of the study products. Mean ± SEM. (■ = test product; ▲ = reference product).

Fig. 1 shows concentration curves for curcumin, curcumin sulfate, and curcumin glucuronide in plasma samples over time. Since all substances washed out so quickly, data presentation for graphing the curves was truncated at 12.0 h.

Concentration of free curcumin from the test product reached a peak at 85 ng/mL ± 0.16 SEM in plasma 1 h after administration (Fig. 1a). Free curcumin from the reference product was not detectable. Concentrations of both the sulfate conjugate (902.36 ng/mL ± 306.98 SEM) and the glucuronide conjugate (10.43 ng/mL ± 3.15 SEM) from the test product peaked at 2 h (Fig. 1b and c). Conjugates from the reference product of sulfate (18.46 ± 10.49 SEM) and glucuronide (0.98 ng/mL ± 0.33 SEM) each reached peak concentration at 5 h.

### 3.2 Bioavailability

Table 2 lists the bioavailabilities of curcumin and its conjugates in the test product in comparison with the reference product. Calculations were derived by converting ng·h/mL from all AUC_0-T_ data to nmol·h/mL.

**Table 2:** Total bioavailability of curcumin and its conjugates after a single oral administration of the test product vs. the reference product.

Constituent	AUC_0-T_ Test product	AUC_0-T_ Reference product	F%
	(nmol·hr/mL ± SEM)	(nmol·hr/mL ± SEM)	
Curcumin	0.0045 ± 0.0013	n/a	–
Curcumin sulfate	7.2657 ± 2.1907	0.4078 ± 0.1177	17,817
Curcumin glucuronide	0.0552 ± 0.0166	0.0045 ± 0.0013	12,267
TOTAL	7.3254	0.4123	17,767

**Notes:** F% = (Total AUC_0-T_ test product/Total AUC_0-T_ reference product) x 100.

**Abbreviations:** F%, percent bioavailability; AUC area under the curve.

### 3.3 Product safety

Safety of the test product and the reference product was assessed by the incidences of adverse events, laboratory tests (hematology, urinalysis, and blood biochemistry), vital signs evaluation, ECG results, and physical examination.

Three adverse events occurred in 2 subjects during the first test period after taking the reference product. All adverse events were of Grade 1 [31]. The 3 adverse events were “possibly related” to the reference product. No action was taken. No subjects dropped out of the study due to adverse events.

No adverse events were observed after subjects took the test product.

No clinically significant changes in lab tests or vital signs occurred in any subject after administration of either the test product or the reference product.

## 4 Discussion

This study confirms the following about the oral administration of 1600 mg of CUMIMUP60® vs. the 95% curcumin reference product: 1) glucuronide and sulfate conjugates predominate in plasma; and, 2) the new formulation vastly improves the bioavailability of curcumin over that of the reference product.

These results also confirm the widely known low absorbability of orally administered free curcumin. The high rate of curcumin conjugation observed here corroborates what has been documented in previous studies [32,33,34,35]. This trend continues even when dosages are much higher, up to 12 g in a single dose [29,36].

Previous comparative pharmacokinetic studies reveal the bioavailability of curcumin to be typically much higher in formulations designed to enhance absorption [37,38,39,40,41,42,43,44].

Several of these enhanced-absorption preparations are commercially available under different registered brand names. Bioavailabilities of curcumin in human studies of example branded products are presented in Table 3 [19,22,42],[45,46,47,48,49],[49,50,51,52,53,54,55,56],.

**Table 3:** Comparison of reported curcumin bioavailabilities among commercial preparations in healthy human subjects.

Product	Increased Relative Bioavailability	Reference
CUMINUP60®	178-fold	This study
BioCurc®	94-fold	[45]
BCM-95®	6.9-fold	[46]
CAVACURMIN®	40-fold	[47]
Curcugen®	49.5-fold	[48]
Curcumin C3 Complex®	1.1-fold	[49]
curcuRouge®	91.8-fold^a^	[50]
CurcuWin®	45.9-fold	[51]
Cureit®	10-fold	[52]
CurQfen®	45.5-fold	[53]
Longvida®	100-fold	[54]
Meriva®	29-fold	[55]
MicroActive® Curcumin SR	9.7-fold	[19]
NovaSOL®	185-fold	[42]
Theracurmin®	27-fold	[22]
TurmiPure Gold®	24.2-fold	[49]
TurmXTRA® 60 N	10-fold	[56]

a Calculated based on comparison with Theracurmin®.

BioCurc® is a liquid droplet micellar formulation of curcumin. It has been shown to raise bioavailability of curcumin by 94-fold [45]. BCM-95® is a mixture of turmeric essential oils and curcumin. Its bioavailability was shown to be 6.9-fold greater than that of standard curcumin [46]. CAVACURMIN® consists of curcumin formulated in a γ-cyclodextrin matrix. It was discovered to boost bioavailability of curcumin by 40-fold over a non-formulated curcumin extract. Curcugen® is a preparation of curcumin in a turmeric oleoresin/essential oils matrix. It has been shown to increase relative bioavailability of total curcuminoids by 49.5-fold [48]. Curcumin C3 Complex® is a standardized curcuminoid extract combined with piperine (Bioperine®). The most recently reported relative bioavailability of curcumins combined with piperine was comparatively low at 1.1-fold [49]. This level contrasts with results of an earlier study showing a curcumin-piperine combination to boost relative bioavailability by 20-fold [21]. CurcuRouge® is made from melted standard curcumin that has been coarsely ground and mixed with modified starch. Although its relative bioavailability has not been directly evaluated, the 91.8-fold increase cited in Table 3 derives from a comparison study with Theracurmin® [50], whose relative bioavailability has been reported [22]. CurcuWin® consists of curcumin enhanced for water-solubility with the hydrophilic carrier, polyvinyl pyrrolidone. It has shown a 49.5-fold increase in relative bioavailability over standard curcumin [51]. Cureit® was developed by formulating a curcumin complex with a polar-non-polar sandwich technology [57]. It has boosted curcumin bioavailability by 10-fold [52]. CurQfen® is a combination of curcumin and a galactomannan-rich fiber extract from fenugreek seeds. It has been shown to boost curcumin bioavailability by 45.5-fold [53]. Longvida® uses particle-based technology for preparing curcumin with a mixture of lecithin and phospholipids from soy. It has been shown to boost curcumin bioavailability by 100-fold [54]. Meriva® is a curcumin-phosphatidylcholine complex made with soy lecithin. It has been shown to boost curcumin bioavailablity by 29-fold over standard curcumin [55]. MicroActive® Curcumin SR is a water dispersible formulation containing dispersed micronized curcuminoids in a sustained-release matrix. It has been shown to increase curcumin bioavailability by 9.7-fold [19]. NovaSOL® is a micelle preparation made with a nonionic surfactant, Tween-80. This formulation has boosted curcumin bioavailability 185-fold [42]. Theracurmin® was prepared by homogenizing a gum ghatti polysaccharide-rich extract with an alcohol-extracted curcumin powder. This preparation has enhanced curcumin bioavailability by 27-fold [22]. TurmiPure Gold® is a mixture of turmeric, acacia gum, and sunflower oil, emulsified with soapbark (quillaja) extract. It was recently found to increase relative bioavailability of curcumin by 24.2-fold over standard curcumin [49]. TurmXTRA® 60 N is a proprietary water-dispersible turmeric extract. It has recently been shown to provide a 10-fold boost in bioavailability of curcumin [56].

As these data show, during the past decade researchers have created different formulations with a focus on improving the bioavailability of curcumin for commercialization. As a result, different products reveal a varying range of enhanced bioavailabilities. Few exact comparisons have been published for head-to-head evaluations within the same study [37,39,47,49,50],[58,59,60]. They have been limited to comparing 2–4 products at a time. For the majority of products, comparisons of bioavailabilities among different studies are inexact due to varying study designs, analytical techniques, pharmacokinetic parameters, dosages, and other relevant details of each formulation. In spite of these drawbacks, literature reports reveal clear trends, with several products exhibiting over 100-fold higher bioavailability relative to standard unformulated curcumin [42,54,61] or close it [45,50].

As of this study, we can now add CUMINUP60® to the list of high-performing curcumin formulations. In addition, the extra advantage of the preparation reported here is that it doesn’t involve toxic solvents [26]. As previously determined, this preparation also has a high drug-loading capacity of 65.5%, which improves its aqueous solubility. Furthermore, the solvent-free co-grinding mixture is simple enough to enhance its suitability for industrial production.

Although the small size of this study precludes any formal safety end point analysis or statistical certainty of safety, the safety profile observed here is consistent with previous clinical and preclinical data [32,35,36,46,62]. However, oral administration of both the test product and the reference product were found to be safe for humans under fasting conditions.

Overcoming the barrier of curcumin’s poor bioavailability is a prerequisite for developing effective functional foods and beverages, supplements, and pharmaceuticals [63,64]. Aside from an earlier study of an unbranded beverage containing Theracumin® [65], food, beverage, and supplement manufacturers have not yet taken full advantage of curcumin formulations with well-documented enhanced bioavailabilities. At least a half-dozen such formulations, including CUMINUP60®, hold great promise for future developments of such over-the-counter health consumables [66].

In addition, although free curcumin is widely acknowledged as the biologically active form of curcumin, many studies have begun to focus on the potential roles of Phase II metabolites such as its glucuronide conjugate [67]. For example, curcumin glucuronide has shown potential for direct anti-cancer activity [68] and for the treatment effects of curcumin *in vivo* mediated at NF-κB [67]. Such roles seem to depend on deconjugation to keep free curcumin in blood [69] and bone [70]. These findings may help explain turmeric’s traditional use in reducing inflammation in tissues rich in the β-glucuronidase that drives deconjugation.

Limitations of the current study point to two main areas where future research can bolster what we know about curcumin bioavailability. The first limitation is the lack of determination of additional derivatives of curcumin, including demethoxycurcuin, bisdemethoxycurcumin, dihydrocurcumin, tetrahydrocurcumin, and hexahydrocurcumin. Although these metabolites are not determined in most studies, a need exists for evaluating their levels in plasma in order to better understand the relationships between curcuminoid pharmacokinetics and physiological/pharmacological effects.

The second limitation is not evaluating the effects of the enhanced absorption of curcumin in CUMINUP60® regarding different physiological outcomes. Comparing treatment effects between free curcumin and CUMINUP60® would fulfill that need.

## 5 Conclusions

Curcumin is one of the most investigated natural products. Its health benefits have been well established. Advances in the drug delivery field hold promising strategies for increasing its bioavailability [44]. The results of the present study provide a new strategy building on that promise. We suggest the co-grinding, solvent-free process used here has broad applications for preparing curcumin as a more effective health ingredient in functional foods and beverages and nutraceuticals.

## CRediT author contributions statement

**Chenjing Wang:** Conceived and designed the experiments, Performed the experiments **Xin Jiang:** Conceived and designed the experiments **Xiaolei Zhang:** Conceived and designed the experiments **Yi Xu:** Conceived and designed the experiments **Li Li:** Analyzed and interpreted the data, Wrote the paper **Xin Li:** Analyzed and interpreted the data, Wrote the paper **Shanglong Wang:** Conceived and designed the experiments, Analyzed and interpreted the data **Ping Shi:** Conceived and designed the experiments, Performed the experiments **Xiaomeng Gao:** Conceived and designed the experiments **Zimin Liu:** Conceived and designed the experiments, Contributed reagents, materials, analysis tools or data **W. Dennis Clark:** Analyzed and interpreted the data, Wrote the paper **Yu Cao:** Conceived and designed the experiments, Performed the experiments, Contributed reagents, materials, analysis tools or data.

## Funding

This research was funded by Chenland Nutritionals, Inc. And by the 10.13039/501100018537National Science and Technology Major Project of China, grant #2020ZX09201-018.

## Data availability

The datasets analyzed during the current study are available from the corresponding author upon reasonable request.

## Declaration of competing interest

The authors declare the following financial interests/personal relationships which may be considered as a potential competing interest: CUMINUP60® is the registered trademark name of Chenland Nutritionals, Inc., for the test product used in the present study. Chenland Nutritionals, Inc., had no role in the design of the study; in the collection, analyses, or interpretation of data; in the writing of the manuscript; or in the decision to publish the results.

## References

[1] Ak T., Gülçin I. (2008). Antioxidant and radical scavenging properties of curcumin. Chem. Biol. Interact..

[2] Amalraj A., Pius A., Gopi S., Gopi S. (2016). Biological activities of curcuminoids, other biomolecules from turmeric and their derivatives - a review. J. Tradit. Complement. Med..

[3] Jurenka J.S. (2009). Anti-inflammatory properties of curcumin, a major constituent of Curcuma longa: a review of preclinical and clinical research. Alternative Med. Rev..

[4] Kishimoto A., Imaizumi A., Wada H., Yamakage H., Satoh-Asahara N., Hashimoto T., Hasegawa K. (2021). Newly developed highly bioavailable curcumin formulation, curcuRouge^TM^, reduces neutrophil/lymphocyte ratio in the elderly: a double-blind, placebo-controlled clinical trial. J. Nutr. Sci. Vitaminol..

[5] Kocaadam B., Şanlier N. (2017). Curcumin, an active component of turmeric (Curcuma longa), and its effects on health. Crit. Rev. Food Sci. Nutr..

[6] Kunnumakkara A.B., Bordoloi D., Padmavathi G., Monisha J., Roy N.K., Prasad S., Aggarwal B.B. (2017). Curcumin, the golden nutraceutical: multitargeting for multiple chronic diseases. Br. J. Pharmacol..

[7] Pulido-Moran M., Moreno-Fernandez J., Ramirez-Tortosa C., Ramirez-Tortosa M. (2016). Curcumin and health. Molecules.

[8] Sohn S.I., Priya A., Balasubramaniam B., Muthuramalingam P., Sivasankar C., Selvaraj A., Valliammai A., Jothi R., Pandian S. (2021). Biomedical applications and bioavailability of curcumin - an updated overview. Pharmaceutics.

[9] Fusar-Poli L., Vozza L., Gabbiadini A., Vanella A., Concas I., Tinacci S., Petralia A., Signorelli M.S., Aguglia E. (2020). Curcumin for depression: a meta-analysis. Crit. Rev. Food Sci. Nutr..

[10] Gagliardi S., Morasso C., Stivaktakis P., Pandinic C., Tinelli V., Tsatsakis A., Prosperi D., Hickey M., Corsi F., Cereda C. (2020). Curcumin formulations and trials: what's new in neurological diseases. Molecules.

[11] Lopresti A.L. (2022). Potential role of curcumin for the treatment of major depressive disorder. CNS Drugs.

[12] Ullah F., Gamage R., Sen M.K., Gyengesi E. (2022). The effects of modified curcumin preparations on glial morphology in aging and neuroinflammation. Neurochem. Res..

[13] Huminiecki L., Horbańczuk J., Atanasov A.G. (2017). The functional genomic studies of curcumin. Semin. Cancer Biol..

[14] Bergonzi M., Hamdouch R., Mazzacuva F., Isacchi B., Bilia A. (2014). Optimization, characterization and in vitro evaluation of curcumin microemulsions. LWT - Food Sci. Technol. (Lebensmittel-Wissenschaft -Technol.).

[15] Cui J., Zhou J., Huang L., Jing J., Wang N., Wang L. (2019). Curcumin encapsulation and protection based on lysozyme nanoparticles. Food Sci. Nutr..

[16] Ji H., Tang J., Li M., Ren J., Zheng N., Wu L. (2016). Curcumin-loaded solid lipid nanoparticles with Brij78 and TPGS improved in vivo oral bioavailability and in situ intestinal absorption of curcumin. Drug Deliv..

[17] Letchford K., Liggins R., Burt H. (2008). Solubilization of hydrophobic drugs by methoxy poly (ethylene glycol)-block-polycaprolactone diblock copolymer micelles: theoretical and experimental data and correlations. J. Pharmaceut. Sci..

[18] Ma Z., Shayeganpour A., Brocks D.R., Lavasanifar A., Samuel J. (2007). High-performance liquid chromatography analysis of curcumin in rat plasma: application to pharmacokinetics of polymeric micellar formulation of curcumin. Biomed. Chromatogr..

[19] Madhavi D., Kagan D. (2014). Bioavailability of a sustained release formulation of curcumin. Integr. Med..

[20] Maiti K., Mukherjee K., Gantait A., Saha B.P., Mukherjee P.K. (2007). Curcumin–phospholipid complex: preparation, therapeutic evaluation and pharmacokinetic study in rats. Int. J. Pharm..

[21] Shoba G., Joy D., Joseph T., Majeed M., Rajendran R., Srinivas P.S. (1998). Influence of piperine on the pharmacokinetics of curcumin in animals and human volunteers. Planta Med..

[22] Sasaki H., Sunagawa Y., Takahashi K., Imaizumi A., Fukuda H., Hashimoto T., Wada H., Katanasaka Y., Kakeya H., Fujita M., Hasegawa K., Morimoto T. (2011). Innovative preparation of curcumin for improved oral bioavailability. Biol. Pharm. Bull..

[23] Yan Y.-D., Kim J.A., Kwak M.K., Yoo B.K., Yong C.S., Choi H.-G. (2011). Enhanced oral bioavailability of curcumin via a solid lipid-based self-emulsifying drug delivery system using a spray-drying technique. Biol. Pharm. Bull..

[24] You J., Dai D., He W., Li G., Song S.C., Wei Y.H., Li F.Z., Xu X.L. (2014). Preparation of curcumin-loaded long-circulating liposomes and its pharmacokinetics in rats. Zhongguo Zhongyao Zazhi.

[25] Zou L., Zheng B., Liu W., Liu C., Xiao H., McClements D.J. (2015). Enhancing nutraceutical bioavailability using excipient emulsions: influence of lipid droplet size on solubility and bioaccessibility of powdered curcumin. J. Funct.Foods.

[26] Lu Y., Lin M., Zong J., Zong L., Zhao Z., Wang S., Zhang Z., Han M. (2020). Highly bioavailable curcumin preparation with a co-grinding and solvent-free process. Food Sci. Nutr..

[27] Asai A., Miyazawa T. (2000). Occurrence of orally administered curcuminoid as glucuronide and glucuronide/sulfate conjugates in rat plasma. Life Sci..

[28] Pan M.H., Huang T.M., Lin J.K. (1999). Biotransformation of curcumin through reduction and glucuronidation in mice. Drug Metab. Dispos..

[29] Vareed S.K., Kakarala M., Ruffin M.T., Crowell J.A., Normolle D.P., Djuric Z., Brenner D.E. (2008). Pharmacokinetics of curcumin conjugate metabolites in healthy human subjects. Cancer Epidemiol. Biomarkers Prev..

[30] Jawień W. (2014). Searching for an optimal AUC estimation method: a never-ending task?. J. Pharmacokinet. Pharmacodyn..

[31] Yasinskaya Y. (2014). Food and Drug Administration, U.S. Department of Health and Human Services.

[32] Cheng A.L., Hsu C.H., Lin J.K., Hsu M.M., Ho Y.F., Shen T.S., Ko J.Y., Lin J.T., Lin B.R., Ming-Shiang W., Yu H.S., Jee S.H., Chen G.S., Chen T.M., Chen C.A., Lai M.K., Pu Y.S., Pan M.H., Wang Y.J., Tsai C.C., Hsieh C.Y. (2001). Phase I clinical trial of curcumin, a chemopreventive agent, in patients with high-risk or pre-malignant lesions. Anticancer Res..

[33] Ireson C., Orr S., Jones D.J., Verschoyle R., Lim C.K., Luo J.L., Howells L., Plummer S., Jukes R., Williams M., Steward W.P., Gescher A. (2001). Characterization of metabolites of the chemopreventive agent curcumin in human and rat hepatocytes and in the rat in vivo, and evaluation of their ability to inhibit phorbol ester-induced prostaglandin E2 production. Cancer Res..

[34] Kunati S.R., Yang S., William B.M., Xu Y. (2018). An LC-MS/MS method for simultaneous determination of curcumin, curcumin glucuronide and curcumin sulfate in a phase II clinical trial. J. Pharmaceut. Biomed. Anal..

[35] Sharma R.A., Gescher A.J., Steward W.P. (2005). Curcumin: the story so far. Eur. J. Cancer.

[36] Lao C.D., Ruffin IV M.T., Normolle D., Heath D.D., Murray S.I., Bailey J.M., Boggs M.E., Crowell J., Rock C.L., Brenner D.E. (2006). Dose escalation of a curcuminoid formulation. BMC Compl. Alternative Med..

[37] Douglass B.J., Clouatre D.L. (2015). Beyond yellow curry: assessing commercial curcumin absorption technologies. J. Am. Coll. Nutr..

[38] Grilc N.K., Sova M., Kristl J. (2021). Drug delivery strategies for curcumin and other natural nrf2 modulators of oxidative stress-related diseases. Pharmaceutics.

[39] Hundshammer C., Schön C., Kimura M., Furune T., Terao K., Elgeti D., Mohr R. (2021). Enhanced metabolic bioavailability of tetrahydrocurcumin after oral supplementation of a γ-cyclodextrin curcumin complex. J. Funct. Foods.

[40] Kocher A., Bohnert L., Schiborr C., Frank J. (2016). Highly bioavailable micellar curcuminoids accumulate in blood, are safe and do not reduce blood lipids and inflammation markers in moderately hyperlipidemic individuals. Mol. Nutr. Food Res..

[41] Prasad S., Tyagi A.K., Aggarwal B.B. (2014). Recent developments in delivery, bioavailability, absorption and metabolism of curcumin: the golden pigment from golden spice. Cancer Res. Treat..

[42] Schiborr C., Kocher A., Behnam D., Jandasek J., Toelstede S., Frank J. (2014). The oral bioavailability of curcumin from micronized powder and liquid micelles is significantly increased in healthy humans and differs between sexes. Mol. Nutr. Food Res..

[43] Stohs S.J., Chen O., Ray S.D., Ji J., Bucci L.R., Preuss H.G. (2020). Highly bioavailable forms of curcumin and promising avenues for curcumin-based research and application: a review. Molecules.

[44] Tabanelli R., Brogi S., Calderone V. (2021). Improving curcumin bioavailability: current strategies and future perspectives. Pharmaceutics.

[45] Stohs S.J., Ji J., Bucci L.R., Preuss H.G. (2018). A comparative pharmacokinetic assessment of a novel highly bioavailable curcumin formulation with 95% curcumin: a randomized, double-blind, crossover study. J. Am. Coll. Nutr..

[46] Antony B., Merina B., Iyer V.S., Judy N., Lennertz K., Joyal S. (2008). A pilot cross-over study to evaluate human oral bioavailability of BCM-95CG (biocurcumax), A novel bioenhanced preparation of curcumin. Indian J. Pharmaceut. Sci..

[47] Purpura M., Lowery R.P., Wilson J.M., Mannan H., Münch G., Razmovski-Naumovski V. (2018). Analysis of different innovative formulations of curcumin for improved relative oral bioavailability in human subjects. Eur. J. Nutr..

[48] Panda S.K., Nirvanashetty S., Missamma M., Jackson-Michel S. (2021). The enhanced bioavailability of free curcumin and bioactive-metabolite tetrahydrocurcumin from a dispersible, oleoresin-based turmeric formulation. Medicine.

[49] Fança-Berthon P., Tenon M., Bouter-Banon S.L., Manfré A., Maudet C., Dion A., Chevallier H., Laval J., van Breemen R.B. (2021). Pharmacokinetics of a single dose of turmeric curcuminoids depends on formulation: results of a human crossover study. J. Nutr..

[50] Sunagawa Y., Miyazaki Y., Funamoto M., Shimizu K., Shimizu S., Nurmila S., Katanasaka Y., Ito M., Ogawa T., Ozawa-Umeta H., Hasegawa K., Morimoto T. (2021). A novel amorphous preparation improved curcumin bioavailability in healthy volunteers: a single-dose, double-blind, two-way crossover study. J. Funct.Foods.

[51] Jäger R., Lowery R.P., Calvanese A.V., Joy J.M., Purpura M., Wilson J.M. (2014). Comparative absorption of curcumin formulations. Nutr. J..

[52] Gopi S., George R., Thomas M., Jude S. (2015). A pilot cross-over study to assess the human bioavailability of “Cureit” a bioavailable curcumin in complete natural matrix. Asian J. Pharmaceut. Technol. Innovat..

[53] Kumar D., Jacob D., Subash P.S., Maliakkal A., Johannah N.M., Kuttan R., Maliakel B., Konda V., Krishnakumar I.M. (2016). Enhanced bioavailability and relative distribution of free (unconjugated) curcuminoids following the oral administration of a food-grade formulation with fenugreek dietary fibre: a randomised double-blind crossover study. J. Funct.Foods.

[54] Gota V.S., Maru G.B., Soni T.G., Gandhi T.R., Kochar N., Agarwal M.G. (2010). Safety and pharmacokinetics of a solid lipid curcumin particle formulation in osteosarcoma patients and healthy volunteers. J. Agric. Food Chem..

[55] Cuomo J., Appendino G., Dern A.S., Schneider E., McKinnon T.P., Brown M.J., Togni S., Dixon B.M. (2011). Comparative absorption of a standardized curcuminoid mixture and its lecithin formulation. J. Nat. Prod..

[56] Thanawala S., Shah R., Alluri K.V., Somepalli V., Vaze S., Upadhyay V. (2021). Comparative bioavailability of curcuminoids from a water-dispersible high curcuminoid turmeric extract against a generic turmeric extract: a randomized, cross-over, comparative, pharmacokinetic study. J. Pharm. Pharmacol..

[57] Amalraj A., Jude S., Varma K., Jacob J., Gopi S., Oluwafemi O.S., Thomas S. (2017). Preparation of a novel bioavailable curcuminoid formulation (Cureit™) using Polar-Nonpolar-Sandwich (PNS) technology and its characterization and applications. Materials Sci. Eng. C - Materials Biol. App..

[58] Gopi S., Jacob J., Varma K., Jude S., Amalraj A., Arundhathy C.A., George R., Sreeraj T.R., Divya C., Kunnumakkara A.B., Stohs S.J. (2017). Comparative oral absorption of curcumin in a natural turmeric matrix with two other curcumin formulations: an open-label parallel-arm study. Phytother Res..

[59] Kothaplly S., Alukapally S., Nagula N., Maddela R. (2022). Superior bioavailability of a novel curcumin formulation in healthy humans under fasting conditions. Adv. Ther..

[60] Stohs S.J., Chen C., Preuss H.G., Ray S.D., Bucci L.R., Ji J., Ruff K.J. (2019). The fallacy of enzymatic hydrolysis for the determination of bioactive curcumin in plasma samples as an indication of bioavailability: a comparative study. BMC Compl. Alternative Med..

[61] Jamwal R. (2018). Bioavailable curcumin formulations: a review of pharmacokinetic studies in healthy volunteers. J. Integrat. Med..

[62] Sharma R.A., Euden S.A., Platton S.L., Cooke D.N., Shafayat A., Hewitt H.R., Marczylo T.H., Morgan B., Hemingway D., Plummer S.M., Pirmohamed M., Gescher A.J., Steward W.P. (2004). Phase I clinical trial of oral curcumin: biomarkers of systemic activity and compliance. Clin. Cancer Res..

[63] Dei Cas M., Ghidoni R. (2019). Dietary curcumin: correlation between bioavailability and health potential. Nutrients.

[64] Munekata P.E.S., Pateiro M., Zhang W., Dominguez R., Xing L., Fierro E.M., Lorenzo J.M. (2021). Health benefits, extraction and development of functional foods with curcuminoids. J. Funct.Foods.

[65] Morimoto T., Sunagawa Y., Katanasaka Y., Hirano S., Namiki M., Watanabe Y., Suzuki H., Doi O., Suzuki K., Yamauchi M., Yokoji T., Miyoshi-Morimoto E., Otsuka Y., Hamada T., Imaizumi A., Nonaka Y., Fuwa T., Teramoto T., Kakeya H., Wada H., Hasegawa K. (2013). Drinkable preparation of Theracurmin exhibits high absorption efficiency - a single-dose, double-blind, 4-way crossover study. Biol. Pharm. Bull..

[66] Tripathy S., Verma D.K., Thakur M., Patel A.R., Srivastav P.P., Singh S., Gupta A.K., Chávez-González M.L., Aguilar C.N., Chakravorty N., Verma H.K., Utama G.L. (2021). Curcumin extraction, isolation, quantification and its application in functional foods: a review with a focus on immune enhancement activities and COVID-19. Front. Nutr..

[67] Bolger G.T., Pucaj K., Minta Y.O., Sordillo P. (2022). Relationship between the in vitro efficacy, pharmacokinetics and in vivo efficacy of curcumin. Biochem. Pharmacol..

[68] Ozawa-Umeta H., Kishimoto A., Imaizumi A., Hashimoto T., Asakura T., Kakeya H., Kanai M. (2020). Curcumin β-D-glucuronide exhibits anti-tumor effects on oxaliplatin-resistant colon cancer with less toxicity in vivo. Cancer Sci..

[69] Ozawa H., Imaizumi A., Sumi Y., Hashimoto T., Kanai M., Makino Y., Tsuda T., Takahashi N., Kakeya H. (2017). Curcumin β-D-glucuronide plays an important role to keep high levels of free-form curcumin in the blood. Biol. Pharm. Bull..

[70] Kunihiro A.G., Luis P.B., Brickey J.A., Frye J.B., Chow H.S., Schneider C., Funk J.L. (2019). Beta-glucuronidase catalyzes deconjugation and activation of curcumin-glucuronide in bone. J. Nat. Prod..

